# Supplementary material for: Cryopreservation and revival of Hawaiian stony corals using isochoric vitrification
Source: Nat Commun. 2023 Aug 23;14:4859. doi: 10.1038/s41467-023-40500-w (PMC10447501; doi:10.1038/s41467-023-40500-w)
Supplement: Supplementary file 3 — Description of Additional Supplementary Files [file 41467_2023_40500_MOESM3_ESM.pdf]

Description of Additional Supplementary Files

File Name: Supplementary Code

Description: This file includes two MATLAB scripts, "fit_resp_data.m" and "resp_ttest2", which enable exponential fitting of raw respirometry data and statistical comparison of exponential fit parameters, respectively. Further detail is provided by comment within each script.
