# Supplementary material for: Cryopreservation and revival of Hawaiian stony corals using isochoric vitrification
Source: Nat Commun. 2023 Aug 23;14:4859. doi: 10.1038/s41467-023-40500-w (PMC10447501; doi:10.1038/s41467-023-40500-w)
Supplement: Supplementary file 5 — Reporting Summary [file 41467_2023_40500_MOESM5_ESM.pdf]

nature portfolio

Corresponding author(s): Matthew J. Powell-Palm, E. Michael Henley

Last updated by author(s): 7/7/2023

Reporting Summary

Nature Portfolio wishes to improve the reproducibility of the work that we publish. This form provides structure for consistency and transparency in reporting. For further information on Nature Portfolio policies, see our Editorial Policies and the Editorial Policy Checklist.

Statistics

For all statistical analyses, confirm that the following items are present in the figure legend, table legend, main text, or Methods section.

n/a	Confirmed	
☐	☒	The exact sample size (n) for each experimental group/condition, given as a discrete number and unit of measurement
☐	☒	A statement on whether measurements were taken from distinct samples or whether the same sample was measured repeatedly
☐	☒	The statistical test(s) used AND whether they are one- or two-sided<br>*Only common tests should be described solely by name; describe more complex techniques in the Methods section.*
☒	☐	A description of all covariates tested
☐	☒	A description of any assumptions or corrections, such as tests of normality and adjustment for multiple comparisons
☐	☒	A full description of the statistical parameters including central tendency (e.g. means) or other basic estimates (e.g. regression coefficient) AND variation (e.g. standard deviation) or associated estimates of uncertainty (e.g. confidence intervals)
☐	☒	For null hypothesis testing, the test statistic (e.g. F, t, r) with confidence intervals, effect sizes, degrees of freedom and P value noted<br>*Give P values as exact values whenever suitable.*
☒	☐	For Bayesian analysis, information on the choice of priors and Markov chain Monte Carlo settings
☒	☐	For hierarchical and complex designs, identification of the appropriate level for tests and full reporting of outcomes
☒	☐	Estimates of effect sizes (e.g. Cohen's d, Pearson's r), indicating how they were calculated

Our web collection on statistics for biologists contains articles on many of the points above.

Software and code

Policy information about availability of computer code

Data collection	No custom code was employed in data collection. Pressure data was acquired using ESI-USB software (no version number available), which is a data acquisition software that accompanies the ESI pressure sensors employed in this study.
Data analysis	All data and statistical analyses were performed in MATLAB 2022b, using built-in statistical analysis and data visualization functions.

For manuscripts utilizing custom algorithms or software that are central to the research but not yet described in published literature, software must be made available to editors and reviewers. We strongly encourage code deposition in a community repository (e.g. GitHub). See the Nature Portfolio guidelines for submitting code & software for further information.

Data

Policy information about availability of data

All manuscripts must include a data availability statement. This statement should provide the following information, where applicable:

- Accession codes, unique identifiers, or web links for publicly available datasets
- A description of any restrictions on data availability
- For clinical datasets or third party data, please ensure that the statement adheres to our policy

All data available upon reasonable request to the corresponding authors. All data presented in the study is also provided in the accompanying Source Data file. This includes all of the both raw and fitted coral respirometry data. Because this raw data is included within the Source Data file and will be archived as such by Nature Communications, we did not think it necessary to host the data on an additional platform.

Human research participants

Policy information about studies involving human research participants and Sex and Gender in Research.

Reporting on sex and gender	N/A
Population characteristics	N/A
Recruitment	N/A
Ethics oversight	N/A

Note that full information on the approval of the study protocol must also be provided in the manuscript.

Field-specific reporting

Please select the one below that is the best fit for your research. If you are not sure, read the appropriate sections before making your selection.

- [x] Life sciences
- [ ] Behavioural & social sciences
- [ ] Ecological, evolutionary & environmental sciences

For a reference copy of the document with all sections, see nature.com/documents/nr-reporting-summary-flat.pdf

Life sciences study design

All studies must disclose on these points even when the disclosure is negative.

Sample size	There was no prior information available on a study such as this to use as a guide for a power analysis. We expect that the large genotype sample numbers should capture most of the standing genotypic variation, and the large number of total replicates used across the treatments should capture that variation as well. Baums et al. 2019 (https://doi.org/10.1002/eap.1978) states that approx. 12 genotypes are sufficient to capture 90% of variation, and we thus used that number as our minimum genotype target for this study.
Data exclusions	No outliers or other data points were excluded.
Replication	No replication issues were encountered in conducting this study, as evidenced by the large trial sizes. To enable efficient replication by other labs in the future, in addition to the description of the isochoric process in the Methods, we have added a more detailed step-by-step "recipe-style" process in the Supplemental Information to add further clarity with the goal of replication in mind. For the microfragmentation and prepping of corals, we have added citations to the Methods that detail those processes. However, it should be noted that the exact location of each parent coral colony on the reef was not tracked for this study, so selecting the exact same coral colonies (these exact genotypes) for a follow-up would not be possible.<br><br>For these experiments, we processed 1-2 genotypes per week (replicated these experiments) over approx. 6 months (July-Dec 2022).
Randomization	For each genotype of coral used, microfragments of that genotype passed through all treatments – with the exception of the positive and negative controls for respirometry analysis. More genotypes / replicates were used for those two groups to provide a robust framework of expected upper and lower boundaries. Individual fragments assigned to treatments groups were selected at random.
Blinding	Blinding was not possible for this experimental design. Corals had to be tracked through each treatment, and there were not sufficient resources to set up a single blind methodology.

Reporting for specific materials, systems and methods

We require information from authors about some types of materials, experimental systems and methods used in many studies. Here, indicate whether each material, system or method listed is relevant to your study. If you are not sure if a list item applies to your research, read the appropriate section before selecting a response.

Materials & experimental systems

n/a	Involved in the study
☒	☐ Antibodies
☒	☐ Eukaryotic cell lines
☒	☐ Palaeontology and archaeology
☐	☒ Animals and other organisms
☒	☐ Clinical data
☒	☐ Dual use research of concern

Methods

n/a	Involved in the study
☒	☐ ChIP-seq
☒	☐ Flow cytometry
☒	☐ MRI-based neuroimaging

Animals and other research organisms

Policy information about studies involving animals; ARRIVE guidelines recommended for reporting animal research, and Sex and Gender in Research

Laboratory animals	No laboratory animals were used
Wild animals	Small fragments of the coral species Porites compressa were collected from large (> 1 meter diameter) wild colonies in near shore reefs close to the study site. These fragments were then trimmed into smaller (1 cm x 1 cm) "microfragments" for use in these experiments. The remaining portions of the colonies that were unused were returned to the reef.
Reporting on sex	The sex of the coral colonies was irrelevant to this study. While most stony corals are hermaphrodites, P. compressa is a gonochore, with colonies broadcast spawning either eggs or sperm a few nights per year in the summer months. There is no sexual dimorphism present in corals; therefore, to determine sex, histological analysis of colonies used would have added thousands of dollars and a year or more of extra analysis.
Field-collected samples	After colony fragments were collected from the reef they were housed in flow-through mesocosms at the marine lab station – natural seawater flowed into the tanks from the bay (from where the parent colonies were collected) that maintained temperature (24-27 C) and were exposed to natural sunlight. Shade cloths were placed over the mesocosms to keep the PAR within a similar range to reef values, and air pumps and powerheads provided water circulation. Coral microfragments used in this study were consumed in the experiments. Remaining fragments not used in experiments were returned to the reef.
Ethics oversight	Fragments were collected under the permits SAP 2022-22 and SAP 2023-31 that were issued by the State of Hawaiʻi Department of Land and Natural Resources. Corals are not subject to IACUC, but the highest standard of husbandry of collected corals was prioritized so that the corals were in a healthy, robust state prior to experimentation.

Note that full information on the approval of the study protocol must also be provided in the manuscript.
